# Research progress on the immune mechanism of thyroid-related eye disease combined with dry eye

**DOI:** 10.1186/s12348-025-00477-7

**Published:** 2025-03-10

**Authors:** Yuhong Wang, Dan Chen, Lina Jin, Dongping Li

**Affiliations:** Aier Eye Hospital of Wuhan University (Hankou Aier), WuHan, HuBei 430000 China

**Keywords:** Thyroid associated eye disease, Dry eye, Immunity, Research progress

## Abstract

Thyroid associated eye disease combined with dry eyes is a common clinical disease and has gradually received clinical attention. Its onset is caused by various factors such as eyelid dynamic change and immune inflammation, involving tear secretion, tear discharge, tear quality and other aspects. This article systematically reviews the research related to its immune factors.

## Introduction

Dry eye is one of the most common ophthalmic diseases, affecting millions of people worldwide. In recent years, it has become an increasingly important health care issue. Studies have shown that dry eye is particularly common in patients with thyroid diseases. About 65-85% of patients with thyroid associated ophthalmopathy (TAO) have dry eye [1–3]. At present, some scholars have invested in the study of the relationship between dry eyes and TAO. Studies have shown that Asians, women (especially postmenopausal women), the elderly, autoimmune diseases, and thyroid dysfunction are risk factors for dry eyes [4]. Dry eyes are one of the most common ocular signs in TAO patients with ocular surface damage. Most patients will experience discomfort symptoms such as dry eyes, foreign body sensation, photophobia and tearing [5–7].

Dry eyes are not simply caused by a decrease in tear volume. Abnormal tear quality also plays an important role in the occurrence of dry eyes [8, 9]. The innermost mucus layer of tears is secreted by conjunctival goblet cells, the aqueous layer is secreted by the lacrimal glands and accessory lacrimal glands, and the lipid layer is secreted by the meibomian glands. The lacrimal glands and their ducts, cornea, tear film, conjunctival and meibomian glands serve as a lacrimal functional unit to maintain the integrity and function of the ocular surface. Any abnormality in any subfunctional unit component can lead to the destruction of tear balance, and the steady-state environment cannot be maintained, resulting in dry eyes or tearing. Relevant studies have shown that the occurrence of TAO dry eye is closely related to increased ocular surface exposure and immune-inflammatory factors. Ocular surface exposure factors mainly include proptosis, eyelid retraction and lag, incomplete eyelid closure, etc. Immune-inflammatory damage factors run through all components of the lacrimal gland functional unit [10–12]. This study focuses on the perspective of immune-related factors and reviews their relationship with the occurrence of dry eye.

### Lacrimal gland

Some studies have shown that the lacrimal gland of TAO patients are also affected, suggesting that lacrimal gland as tear secretion organs, may be an important cause of TAO combined with dry eyes [13, 14]. Eckstein et al. [15] first reported that the lacrimal gland acinar cells of normal people express thyroid stimulating hormone receptors (TSHR). The lacrimal gland of TAO patients become the target of autoantibody attack, resulting in lacrimal gland damage and reduced tear production. Shoaib Ugradar MD [16] observed the changes in the lacrimal gland and tear after teprotumumab treatment of TAO and found that the lacrimal gland volume was reduced and tear secretion increased. Nicole Tsz Yan Wong [17] showed that the coronal and sagittal volumes of the tear glands in the active stage were significantly increased, and concluded that the ocular surface damage caused by tear factors was related to lacrimal gland inflammation. Xulin Liao [18] studied the volume of the lacrimal gland and extraocular muscles of patients with inactive TAO dry eyes through MRI and found that the lacrimal gland volume of patients with inactive TAO with severe subjective symptoms was relatively small, indicating reduced lacrimal gland function and reduced secretion.

### Cornea

During the course of TAO, ocular surface damage is the main cause of dry eye symptoms in patients, and the cornea is the most direct and sensitive effector organ of ocular surface damage. Current studies have found that corneal changes are mainly reflected in corneal inflammation and decreased nerve sensitivity. Canan Gurdal [19] proposed that in TAO, dry eye symptoms and ocular surface damage are most likely related to ocular surface inflammation, and it may be the only manifestation before TAO occurs in patients with abnormal thyroid function. Zhang Hongjuan’s research [20] also showed that before TAO occurs, the patient’s ocular surface has inflammatory damage. Edoardo Villani et al. [21] found through confocal microscopy that the number of activated cells in the cornea increased significantly during the active stage of TAO, and that corneal sensory sensitivity was negatively correlated with exophthalmos. Wu Lian-Qun et al. [22] found that the density and maturity of Langerhans cells in the cornea of TAO patients increased, revealing that the cornea is in an immune-activated state, indicating that the cornea, as a relatively immune-free organ, is still affected in TAO. Mutlu Acar [23] observed that after 1 year of high-dose hormone therapy, the number of corneal inflammatory cells decreased and the tear film stability increased. Symptoms were stable and the quality of life improved for up to 4 years. In addition to the increase in corneal inflammatory cells, some studies have shown that the number of corneal epithelial cells decreased. The coloration of corneal fluorescent staining is also a side reaction. Tear factor detection showed an increase in metalloproteinase MMP-9. It is speculated that MMP-9 dissolves the tight junctions between the epithelial basement membrane and proteins, which is a possible cause of corneal epithelial damage [24].

The cornea is rich in nerve endings, which not only maintain the sensation of the cornea, but also play an important role in ion transport between corneal layers, cell division, wound healing, and maintaining the stability of the ocular surface microenvironment. Corneal sensation is the most prominent feature of corneal nerves, and corneal nerves have a direct effect on the nutrition of the corneal epithelium. Vasilis Achtsidis examined corneal sensation in early active TAO and found that even if the eyeball did not protrude significantly, corneal sensation was significantly reduced, which was related to the occurrence of dry eyes [25]. Achtsidis and Villani et al. pointed out that the corneal sensation of TAO patients was significantly reduced, and the mechanism of corneal nerve ending lesions may be due to the inflammatory response of the ocular surface of TAO patients, which causes damage to the corneal base nerve plexus [21, 25]. The immune inflammatory edema of the extraocular muscles and orbital fat in TAO causes increased orbital pressure. The deep trigeminal nerve sensory branch may be compressed and edematous. On the one hand, this reduces the sensitivity of corneal nerve perception, reduces its nutritional effect on the corneal epithelium and weakens its repair ability. On the other hand, it causes a decrease in blink frequency and increased tear evaporation, causing dry eyes [8].

### Conjunctiva

Conjunctival goblet cells secrete the mucin layer and evenly spreads it on the ocular surface. If the conjunctiva changes, dry eyes will also occur. Impression cytology is often used for cytological studies of the conjunctival surface [26, 27]. The normal conjunctival epithelium includes non-keratinized squamous epithelial cells and goblet cells. Ismailova et al. [9] found that TAO patients had dystrophy of the conjunctival epithelial cells with cell polymorphism, reduced and apoptotic goblet cells, and epithelial keratinization with leukocyte infiltration. The upper eyelid conjunctiva of patients with stable TAO showed no difference from the control group in terms of inflammatory response, fibrosis, macrophage infiltration, and fibroblast quantification, indicating that the upper eyelid conjunctiva can be protected from damage during the course of TAO and can still perform other functions such as secreting tears, thereby reducing ocular surface damage in TAO patients. Yi-Hsuan Wei [28] used confocal microscopy to observe the bulbar conjunctiva and found that the density of Langerhans cells in the bulbar conjunctival epithelium of TAO patients increased, while the density of goblet cells decreased. Impression cytology was used to find obvious squamous metaplasia of the bulbar conjunctiva. The research data of Xu Nuo et al. [29] showed that the distance between conjunctival epithelial cells in TAO patients became larger, the density of goblet cells decreased, the mucin secreted by them decreased, and the conjunctiva in the palpebral fissure area showed 2–3 grade squamous metaplasia, which was consistent with the conclusion of previous scholars. He also pointed out that the pathological process of squamous metaplasia is the loss of local vascularization and the formation of scars. Due to the influence of inflammatory factors, the conjunctival congestion and edema, which is exposed to dryness, leads to a decrease in conjunctival goblet cells and epithelial cells, which further leads to a lack of mucin layer. Mucin changes cause tear film instability, leading to dry eyes.

### Tear film

As the first defense barrier, the tear film covers the surface of the cornea, and the maintenance of its stability is the key to the comfort of the ocular surface. The observation of the tear film rupture time by corneal fluorescein staining is a commonly used index in the clinic to judge the stability of the tear film.The stability of tear film requires the stable composition of tear components and the stable dynamics of tear circulation. Any factor affecting the change of tear composition and tear secretion and discharge may lead to the decrease of the stability of tear film and cause dry eyes.In 1992, A. K. Khurana et al. [30] proposed that in patients with advanced TAO, BUT was significantly shortened and the staining score was high, indicating that the stability of tear film was damaged.Most of the current studies have shown that the tear osmolality is generally increased in patients with TAO.Iskeleli et al. [31] studied osmolality of tears in TAO patients was (340.38 ± 18.74) mOsm increased in healthy people (290.80 ± 13.58)mOsm. This change may be associated with proptosis and eyelid fissure widening in TAO patients, which often leads to ocular surface damage.Research [32, 33] shows that when the ocular surface is in a hypertonic environment for a long time, it can cause the oxidative stress response in the ocular surface cells, the cell DNA damage fracture, and the cell cycle arrest.The abnormal activation of MAPK signaling pathway leads to the increase of the expression of inflammatory factors such as interleukin lineage and tumor necrosis factor, which can promote the maturation and activation of APC cells, and activate Th1 and Th17 mainly T lymphocytes, and then lead to the inflammation.

The changes in tear composition start from the three-layer structure, which are mainly manifested by decreased mucin content, lack of water, increased content of various inflammatory factors and abnormal proteins, and abnormal lipid secretion.Mucin is secreted by conjunctival goblet cells. The decrease in the number and function of conjunctival goblet cells leads to a decrease in the mucin content in tears. TAO patients, especially those in the active stage, often have varying degrees of conjunctival congestion and edema. Some researchers have used conjunctival impression cytology and in vivo confocal microscopy to examine the conjunctiva. The results showed that the conjunctival epithelium had varying degrees of squamous metaplasia, and the density and number of goblet cells decreased. It was also found that the conjunctival inflammatory cells increased [9, 28, 34]. Argueso et al. [35] used ELISA to observe mucin in the tears of patients with dry eye and pointed out that MUC5AC mucin decreased significantly. They also found that the number of goblet cells synthesizing MUC5AC mucin was significantly reduced.

The connection of proteins in tears play an important role in maintaining the tension and stability of the tear film. Normal human tears are rich in protein, including immunoglobulins, lysozyme, anti-inflammatory factors, etc. In recent years, TAO tear proteomics research has provided some new insights into the tear protein components [36–38]. Nina Matheis [39] used matrix-assisted laser desorption ionization mass spectrometry to measure TAO tear protein and found that proline-rich protein 1, uridine diphosphate glucose dehydrogenase, transcriptional regulator BRG1, cystatin, annexin A1, heat shock protein 27 and galectin were down-regulated, while the Midasin protein family and POTEI were up-regulated. The up-regulation and protective down-regulation of inflammatory proteins showed significant differences, which can be used as effective indicators of disease activity and ocular surface diseases. Jiang Lihong et al. [40] found that the expression of lactoferrin and lysozyme C in tears of patients with TAO was increased. Aass et al. [41, 42] tested 1212 proteins in the tears of patients with moderate to severe TAO. The results showed that 16 proteins showed significant differences between the two groups, especially lysozyme C, anti-albumin, lachrymal protein and zinc α2 sugar Protein-1 showed upregulation, which also confirmed that the lacrimal gland is the target affected organ in TAO disease.

Normal human tears contain a variety of cytokines, and the concentration of each cytokine maintains a steady state to regulate cell function. In TAO patients, stimulation of thyroid autoantigens causes changes in corresponding cytokine concentrations, causing orbital tissue edema, lacrimal gland inflammation and damage, rapid tear film evaporation, and increased tear film osmotic pressure [8, 31]. The most commonly studied ones at present are tumor necrosis factor (TNF), interferons (IFN) and interleukin (IL). The results of a study by the Singapore National Eye Center [43] showed that IL-7 levels in the active phase of TAO were significantly different from those in the control group, indicating that IL-7 may have a certain immune repair effect on TAO. Huang Danping et al. [44] used multiplex magnetic bead flow cytometry to observe 8 tear cytokines. The results showed that the levels of IL-1β, IL-6, and IL-8 in TAO patients in the active phase were much higher than those in stable phase and healthy control patients. Uihelyi et al. [45] analyzed the tears of TAO and patients with thyroid dysfunction without ocular manifestations and found that the tears of the two groups contained IL-1β, IL-6, IL-13, IL-17 A, IL-18, and TNF-α. There was no statistical difference in the content of RANTES, indicating that TAO ocular surface damage may precede orbital damage, and ocular surface tissue may be an independent target affected organ in TAO. Jin Sook Yoon et al. [46] have shown that NGF may have a special role in ocular surface inflammation and have a protective effect on ocular surface damage in patients with TAO activity. Anti-inflammatory treatment significantly reduces NGF levels in tears, increases tear film stability and secretion, and reduces congestion symptoms.

### Meibomian glands

Meibomian glands are involved in the secretion of the lipid layer of the tear film. Through intermittent blinking, the secreted lipids are evenly spread over the entire ocular surface, preventing tear evaporation and maintaining the integrity and stability of the tear film. TAO patients often have meibomian gland dysfunction or blepharitis. The myosin in the muscle fibers around the meibomian glands is decomposed, the meibomian gland lipid excretion power is weakened, the complete blinking is reduced, and the distribution of meibomian fat on the ocular surface is reduced, affecting the stability of the tear film [3, 47, 48]. The incidence of meibomian gland dysfunction in TAO patients is increasing [49]. The changes in lipids in meibomian gland dysfunction in patients with dry eye are mainly: increased free fatty acids, wax esters, sterol esters and branched fatty acids, and decreased cholesterol, triglycerides and phospholipids. These changes lead to gland duct blockage. Jinhwan Park [50] used Lipiview to evaluate the lipid layer thickness of TAO patients and found that the average lipid layer thickness was 82.43 ± 24.52 nm. He proposed that increased frequency of incomplete blinking, poor meibomian gland function, and meibomian gland loss are possible causes of TAO dry eyes. Kenneth Ka Hei Lai’s study [3] found that the more severe eye in TAO patients had more severe dry eye and a higher rate of meibomian gland atrophy. Xulin Liao et al. [47] evaluated dry eye in untreated TAO patients and found that meibomian gland dysfunction in the lower eyelid was significant, which together with exophthalmos affected the stability of the tear film and easily led to evaporative dry eye.

## Summary

To sum up, dry eye is a common complication of thyroid-associated ophthalmopathy (TAO) and has received extensive attention. Immune factors causing ocular surface damage play a significant role in its pathogenesis and affect various components of the lacrimal gland functional unit. In addition, the increased exposure rate of the ocular surface plays an important role. In-depth exploration of its complete pathogenesis will provide an important theoretical basis for subsequent treatment. Further research is still needed to improve understanding in this field.

## Data Availability

No datasets were generated or analysed during the current study.
